# Patients' age as a determinant of care received following acute stroke: A systematic review

**DOI:** 10.1186/1472-6963-11-161

**Published:** 2011-07-06

**Authors:** Julie A Luker, Kylie Wall, Julie Bernhardt, Ian Edwards, Karen A Grimmer-Somers

**Affiliations:** 1 International Centre for Allied Health Evidence, University of South Australia, Adelaide, South Australia; 2 Flinders Medical Centre, Bedford Park, South Australia; 3 Physiotherapy School, La Trobe University, Melbourne, Victoria, Australia; 4 Stroke Division, Florey Neurosciences Institute, Heidelberg Heights, Victoria, Australia; 5 School of Health Sciences, University of South Australia, Adelaide, South Australia

## Abstract

**Background:**

Evidence-based care should improve acute stroke outcomes with the same magnitude of effect for stroke patients of all ages. However, there is evidence to suggest that, in some instances, older stroke patients may receive poorer quality care than younger patients.

Our aim was to systematically review evidence of the quality of care provided to patients with acute stroke related to their age. Quality of care was determined by compliance with recommended care processes.

**Methods:**

We systematically searched MEDLINE, CINAHL, ISI Web of Knowledge, Ageline and the Cochrane Library databases to identify publications (1995-2009) that reported data on acute stroke care process indicators by patient age. Data extracted included patient demographics and process indicator compliance. Included publications were critically appraised by two independent reviewers using the Critical Appraisal Skills Programme tool, and a comparison was made of the risk of bias according to studies' findings. The evidence base for reported process indicators was determined, and meta-analysis was undertaken for studies with sufficient similarity.

**Results:**

Nine from 163 potential studies met the inclusion criteria. Of the 56 process indicators reported, eleven indicators were evidence-based. Seven of these indicators (64%) showed significantly poorer care for older patients compared to younger ones, while younger patients received comparatively inferior care for only antihypertensive therapy at discharge. Our findings are limited by the variable methodological quality of included studies.

**Conclusion:**

Patients' age may be a factor in the care they receive after an acute stroke. However, the possible influence of patients' age on clinicians' decision-making must be considered in terms of the many complex issues that surround the provision of optimal care for older patients with acute stroke.

## Background

It has been reported that older patients with an acute stroke have poorer outcomes than younger patients [1]. Age-related differences in co-morbidities, stroke risk factors and stroke severity may contribute to this. However, recent research suggests that when evidence based care is provided, patient outcomes improve with the same magnitude of effect, regardless of age differences [2]. Emerging evidence indicates that poorer outcomes for older patients with stroke may correlate with poorer quality of care [3-5]. Recently there have been significant advances internationally in the standardisation of evidence-based stroke care [6]. Several studies have examined compliance with evidence-based care recommendations for acute stroke, and have identified factors that may result in poorer care [7-11]. One such factor is patient age.

This review aimed to examine whether older acute stroke patients received the same quality of care as younger patients, as measured by compliance with evidence-based process indicators. This is the first known systematic literature review to synthesise evidence of differences in acute stroke care associated with age.

## Methods

The PRISMA Statement underpinned the process of conducting and reporting this review [12].

### Eligibility criteria

Types of studies: Published evidence was sought from studies including systematic reviews and meta-analyses, experimental studies, time series studies, prospective and retrospective observational studies, single case studies and case-control studies. Studies were eligible for inclusion if they described process indicators for health care delivered for patients with acute stroke. The search period of January 1995 to December 2009 was chosen to include the earlier evaluations of organised stroke care, and no language limits were imposed [13].

Types of participants: Adults (over 18 years old) who were admitted to hospitals with acute stroke. All models of acute stroke care were included.

Types of outcome measures: Compliance with process indicators relating to the care received by patients within the first two weeks of hospital admission, reported by age groups.

### Information sources

Computerised bibliographic databases (and platforms) were searched by a single reviewer (JL): MEDLINE (Ovid), CINHAL (EbscoHost), The Cochrane Library Database of Systematic reviews, ISI Web of Knowledge and Ageline (Ovid). The bibliographies of included articles were pearled for additional publications that met inclusion criteria.

### Search

The search used boolean operators to combine free text terms and/or MeSH terms including: stroke; cerebrovascular accident; quality of health care; quality; process indicator; access; health services accessibility. An example of the MEDLINE search strategy appears in Additional file 1.

### Study selection

Titles and abstracts were initially screened by the primary researcher (JL) to eliminate obvious irrelevance. Two reviewers (JL & KW) then independently examined the remaining abstracts against the inclusion criteria in an unblinded, standardised manner. Potentially relevant full text publications were reviewed in detail. In the event that more than one publication had analysed the same data set, the most relevant publication was included. At all stages of the review process, the reviewers discussed opinions and a third independent reviewer was available for arbitration.

### Data collection process

Data were extracted and entered into a purpose-built MS Excel data sheet by one reviewer (JL), and a second reviewer (KGS) checked the data when uncertainties were encountered. The authors of one study were contacted for data clarification to assist with a cluster meta-analysis [7].

### Risk of bias in individual studies

Included studies were appraised by two independent reviewers for their risk of bias and quality of reporting using the Critical Appraisal Skills Programme (CASP) tool designed for cohort study appraisal [14]. This tool provided a 12 point check list of study validity, risk of bias in recruitment, exposure, outcome measurement, confounding factors, reporting of results and the transferability of findings (maximum sore of 12).

### Data extraction

We extracted data on the process indicators reported, the evidence of compliance with these indicators, and compliance of care related to patient age. We also extracted other explanatory data where available, including stroke severity, comorbidity, gender, pre-morbid functional level, stroke unit admission, weekend admission, and the studies' purpose and methodology.

### Risk of bias analysis

A comparison was made of the risk of bias (CASP scores) according to studies' findings. Studies were allocated to two groups:

1. Studies that reported only care favouring younger patients

2. Studies that reported care favouring older patients as well as care favouring younger patients or equivocal results.

Average CASP scores (Standard deviations (SD)) were calculated per group, and differences were assessed using Student t-tests for unequal variance (*p < 0.05)*.

### Data analysis

Evidence-based indicators: Process indicators and their underpinning research evidence were aligned to the Australian Clinical Guidelines for Stroke Management 2010 [15]. This review then focused only on process indicators backed by high level evidence (Level 1 of the Australian National Health & Medical Research Council's Evidence Hierarchy) [16]. Compliance with these process indicators, and age-related differences in compliance were then synthesised and reported descriptively.

Cluster meta-analysis: Meta-analysis was undertaken where there was sufficient similarity between studies regarding participant age groupings and reported process indicators. Studies did not come from a common population, therefore random effects models were developed so that both within- and between-study variability could be considered. MedCalc software was used and the DerSimonian and Laird approach was employed to calculate the summary odds ratio under the random effects model [17,18].

## Results

### Study selection

**Figure 1:**
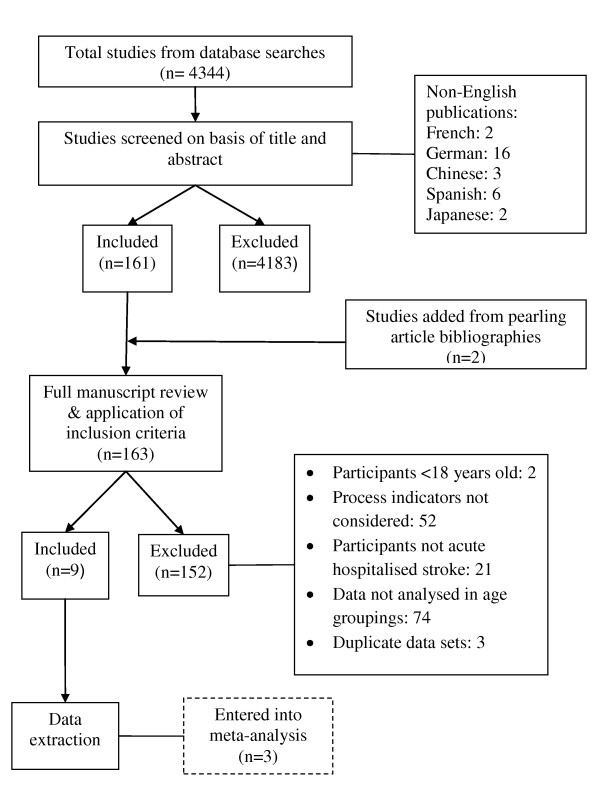
**Flowchart of document identification and selection process**.

The literature identification and selection process is summarised in Figure 1, and the included literature is listed in Table 1. Non-English publications were considered (n = 29), however none met the inclusion criteria at title and abstract screening.

**Table 1:** Comparison of included studies

First author year	Country language	Sample size	Study's design & primary objective	Sample demographics	Age ranges	Confounders considered	Acute care processes indicators audited (Level 1)	CASP quality score
Bhalla 2004 [8]	Europe 10 countries English	1847	Observational, retrospective. To estimate the structure & process of care, and independent factors associated with 3 month mortality and functional outcomes in patients aged >75 yrs compared to younger stroke patients	Mean age 70.2 (SD 12.3) Age range 34-93	<75, ≥75	Age, Gender, Pre-stroke function, Previous home situation, Stroke type, Stroke severity, Stroke unit/other, Comorbidities	CT head scan, Angiography, Stroke unit care, Carotid imaging.	8

Di Carlo 1999 [9]	Europe 7 countries English	4499	Observational, prospective. To evaluate stroke features, hospital resource usage and functional outcome in patients aged ≥80 years compared with the younger age groups.	Mean age 71.8 (SD 12.6) Age range 13-102 Females 50.2% Stroke types: Infarct 60.9% Haemorrhage 10.3%	<80, ≥80	Age, Gender, Pre-stroke function,	CT head scan, Angiography.	7

Fairhead 2006 [20]	UK English	681004	Observational, retrospective. To evaluate the investigation of carotid stenosis in older patients with TIA and stroke.	Nil reported	<80, ≥80	Age, Gender, Stroke type, Degree of stenosis	Angiography, Carotid imaging.	8

Heidrich 2007 [21]	Europe 10 countries English	1721	Observational, retrospective. To investigate variations in use of diagnostic procedures across selected European acute stroke hospitals.	Mean age 69 (SD 12.9) Females 53.6% Stroke types: infarct 57% Haemorrhage 10.3% SU care 14.8%	<65, 65-74, 75-84, ≥85	Age, Stroke severity, Comorbidities, Delay to hospital, Geography	CT head scan.	9

McKevitt 2005 [11]	UK English	1635	Observational, prospective. To determine whether patterns of clinical service provision differ by age, sex, socioeconomic status (SES) or ethnicity	Mean age 71.6 (SD 14.2) Females 51.3%	<65, 65-74, 75-84, ≥85	Age, SES, Gender, Ethnicity, Pre- post-stroke morbidity, Disability, Stroke severity, Stroke unit/other, Cognition	CT head scan Stroke unit care	9

McNaughton 2003 [19]	New Zealand English	181	Observational, prospective. To test whether current measures of stroke processes are related to stroke outcome	Mean age 74.4 (SD 12) Females 53% Stroke types; Infarct 86.1% Haemorrhage 9.9%	<75, >75 (sic)	Age, Ethnicity, Stroke type, Admission function, Discharge function	CT head scan, Swallow screen,	6

Palnum 2008 [10]	Denmark English	29549	Observational, retrospective. To examine the fulfilment of stroke quality-of-care criteria according to age and the possible impact of age-related differences on mortality.	Nil reported	<65, 65-80, >80	Age, Gender, Pre-stroke function, Previous home situation, Stroke severity, Comorbidities	CT head scan, Stroke unit care, Antithrombotic/antiplatelet therapy, Anticoagulants for AF,	10

Rudd 2007 [7]	UK English	8718	Observational, retrospective. To determine whether access to high-quality stroke care is affected by the age or gender of patients, or by weekend admissions	SU care 46%	<65, 65-74, 75-84, ≥85	Age, Gender, Stroke unit/other, Weekend admission	CT head scan, Stroke unit care, Carotid imaging, Asprin commenced early, Antithrombotic therapy by discharge, Risk factor information to patient, Anticoagulants for AF, Antihypertensive therapy, Swallow screen.	9

Saposnik 2009 [22]	Canada English	3631	Observational, prospective. To determine whether access to stroke care, delivery of health services, and clinical outcomes after stroke are affected by age	Mean age 72.0 Females 47.8% Previous NH 5.6% Comorbidities: Low CCI ≤1 65.8%	<59, 60-69, 70-79, >80	Age, Gender, Stroke unit/other, Stroke severity, Comorbidities	Thrombolysis therapy, Stroke unit care, Carotid imaging, Antithrombotic/antiplatelet therapy, Anticoagulants for AF, Antihypertensive therapy, Swallow screen,	10

**Table legend: **SU = stroke unit SD = Standard deviation UK = United Kingdom CCI = Charlson Comorbidity Index

### Study characteristics

Nine studies provided data on age-related differences in quality of care. Table 1 details the selected studies in terms of sample size, country of origin, study objective, study design and reported data. This table illustrates the differences between the studies in terms of categorisations of age. Age 75 years was chosen to differentiate between younger and older patients in two studies [8,19], 80 years was chosen in two studies [9,20], and an assortment of 10 year age ranges were reported in the remaining five studies. All studies had included data from more than one hospital site, and there was no apparent uniformity in the model of stroke care provided across sites. Some participants in each study had access to acute stroke unit care, with the exception of McNaughton's New Zealand study where no hospital sites provided this type of care [19].

### Risk of bias within studies

The CASP Cohort Study appraisal scores varied, although five studies scored nine or more from a possible CASP score of 12. Due to cultural variation and differences in the structure of acute stroke services, none of the studies scored perfectly for transferability of results (see Table 1). The majority of studies had a clearly focused question and used appropriate methodologies. All studies scored poorly on the identification of important confounding factors, including patients' pre-morbid status, co-morbidities and week-end admissions. The fact that some studies included data that was already six to ten years old at the time of publication was a concern [8,9,11,19,21]. The CASP Cohort Study appraisal scores can be found in Additional file 2.

### Results of studies

In total, 56 process indicators for acute stroke care were identified in the included papers. These reflected initial assessment and treatment, management and rehabilitation, secondary prevention and discharge planning. Eleven of these indicators were underpinned by Level 1 evidence (Table 2). The remaining process indicators were assessment for visual fields, level of consciousness, nutritional risk, mood, cognition, allied health discipline-specific assessments, multidisciplinary team meetings, agreed rehabilitation goals, urinary continence plans, elements of patient-centred care, and information provision and various hospital discharge processes. The complete list of process indicators considered by each research group is provided in Additional file 3.

**Table 2:** Reported compliance with evidence-based process indicators for acute stroke care

Process of stroke care	**Palnum 2008**[10]	**Rudd 2007**[7]	**Bhalla 2004**[8]	**Di Carlo 1999**[9]	**Fairhead 2006**[20]	**Heidrich 2007**[21]	**McNaughton 2003**[19]	**Saposnik 2009**[22]	**McKevitt 2005**[11]
CT head scan performed	**Y**	**Y**	**Y**	**Y**		**Y**	**Y**		**Y**

Angiography*			**Y**	**Y**	**Y**				

Thrombolysis therapy								**ND**	

Asprin/antiplatelet therapy commenced early	**Y**	**Y**							

Swallow screen/assessment		**O**					**Y**	**O**	

Care in a stroke unit	**Y**	**Y**	**ND**					**ND**	**ND**

Carotid imaging		**Y**	**Y**	**Y**	**Y**			**Y**	

Stroke risk factors discussed with patient/carer		**Y**							

Antithrombotic treatment by discharge	**ND**	**ND**						**ND**	

Anticoagulant therapy for atrial fibrillation	**Y**							**ND**	

Antihypertensive therapy at discharge								**O**	

Table legend: * includes conventional arterial & venous angiography, computer tomography angiography & magnetic resonance angiography

**Y **= Compliance significantly favours younger patients

**O **= Compliance significantly favours older patients

**ND = **No significant age difference in compliance

**Empty cells **= Process indicator not examined in the reviewed literature

All studies reported at least one process indicator where the care for older patients was significantly less compliant with evidence-based recommendations than care received by younger patients. In two papers, poorer compliance with certain care processes for younger patients compared to older patients, was also identified. Overall, our review found that older patients were disadvantaged, with poorer compliance with seven (64%) of the evidence-based process indicators. In contrast younger patients had poorer compliance with one indicator for antihypertensive therapy on discharge (9%). Although two studies reported inferior care for younger patients for the process of swallow screening, another study showed inferior care for older patients. Age-related findings for this indicator are therefore equivocal (Table 2). Additional data are provided in Additional file 4.

### Synthesis of results

Risk of bias across studies: There was a statistical trend (non-significant) demonstrating a weak relationship between the quality of the study (CASP Score) and findings of age-related differences in care (p = 0.07). The group of studies consistently showing that stroke care which favoured younger patients had an average CASP score of 7.5 (Std Dev 1.3) [9,19-21]. Studies in which there was no consistent age-related care benefit had an average CASP score of 9.2 (Std Dev 0.84) [7,8,10,11,22].

**Figure 2:**
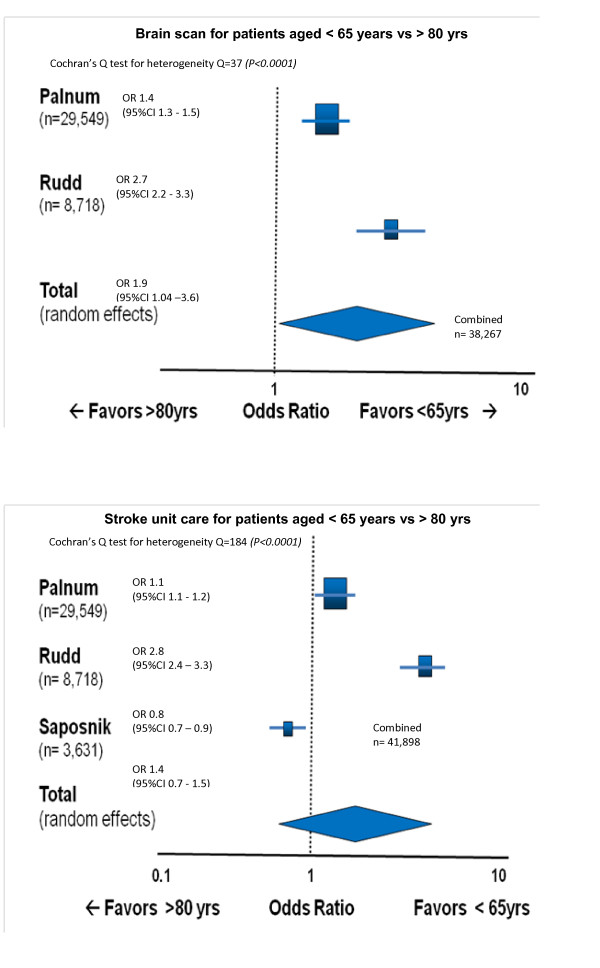
**Meta-analysis results**.

Meta-analysis (Figure 2): Cluster meta-analysis was undertaken with three large studies with sufficient homogeneity in age categorisations and process indicators [7,10,22]. Meta-analysis confirmed that patients over 80 years of age were significantly less likely to receive a timely CT scan after stroke than younger patients. There were equivocal findings however, for admission to a stroke unit. While the Danish study by Palnum's group and Rudd's UK study both found that patients older than 80 years were less likely to receive care in a stroke unit [7,10], the Canadian study by Saposnik et al demonstrated that older patients were more likely to be admitted to a stroke unit than those younger than 65 years [22]. As expected, combining these results in a meta-analysis resulted in the loss of significance of association.

## Discussion

This is the first known systematic review to examine the published literature for age-related differences in the care provided to patients admitted to hospital with acute stroke. This review suggests that there may be age-related decisions made by clinicians regarding care provision, despite strong research underpinning care recommendations applicable to all stroke patients. Age-related differences in care varied across settings, and care was not always systematically biased against older patients. This review raises many important questions regarding how clinicians decide on the provision of optimal care for patients with acute stroke, related to their age.

Complex issues surround clinical care decisions for stroke patients irrespective of their age. These must be considered in the context of the findings of this review. The evidence to guide some care processes for older patients with stroke remains unclear, due to many large intervention studies excluding patients older than 80 years [5,23]. Decisions regarding the best care to provide for many older patients are further complicated by the many factors that accompany ageing, such as increasing comorbidity and disability levels, and stroke severity [24-26].

Methodological concerns in the included studies limited conclusions from this review. To illustrate, the current lack of agreement on the age at which stroke patients were considered 'older' challenged comparison between studies, and also compromised opportunities to pool results. The recent review of stroke management in very old patients by Sanossian and Ovbiagele (2009) suggested that many studies in this field adopt an age cut-off of 80 years or older, however the current lack of standardisation in the literature remains an issue when reporting age-related care [5]. Critical appraisal of study quality highlighted weaknesses that constrained the rigour and credibility of study findings. We found that studies of higher methodological quality showed mixed results. Higher quality studies are less likely to report biased findings caused by Type 1 error [27], therefore there is less certainty in the findings of poorer quality studies that found only care disadvantaging older patients.

Of particular relevance to this review was the lack of consideration given to possible confounding factors, such as weekend admission, which has been associated with sub-optimal quality of stroke care [28]. Other methodological flaws, such as small sample size and the use of very old data, led us to be cautious in drawing conclusions from some study findings. All but two included studies were conducted in Europe. As stroke management systems, as well as attitudes toward older people may differ between cultures, our results may be culturally limited. The review identified a large number of process indicators, but only a proportion of them were evidence-based and linked to improvements in stroke outcomes. Positive outcome publication bias may also influence reviews of this type, due to researchers and journals possible reluctance to publish unfavourable results [29]. We also acknowledge that the recommendations for acute stroke care progressed rapidly over the time period of our review (1995-2009), and some of the variability between studies may reflect historical differences.

Our review suggests an overall pattern of inferior care provided to older stroke patients compared to younger ones. Saposnik and colleagues report that the provision of optimal stroke unit care improves acute stroke outcomes with the same magnitude of effect for stroke patients in all age groups [2]. For patients older than 80 years, the 'numbers needed to treat' (NNT) to improve outcomes through the provision of stroke unit care, are very low (NNT = 5 to prevent one death by 30 days). Five studies examined the gold standard for 'care within a dedicated stroke unit' however, even within this framework there was not universal agreement about older patients' access to stroke unit care (Table 2). There is evidence that care within a stroke unit improves the chances of older patients receiving evidence-based care [2]. This is supported by Rudd and colleagues (2007) who reported on the processes of care met, stratified according to patients' admission to a stroke unit or non-stroke unit. They found that care was consistently better for patients of all ages in a stroke unit [7]. However this was not supported by Bhalla and colleagues (2004) who reported that older patients tended not to receive appropriate care, even if admitted to a stroke unit [8]. Despite the non-significant pooled result from our meta-analysis for this process indicator (Figure 2), important questions are raised by the significant, yet different, age-related findings of the individual studies [7,10,22]. It is possible that the three studies reflect international differences in stroke systems of care, and future research into these differences may provide insights into the best models for promoting equity.

The use of carotid imaging was another frequently considered process indicator that has good evidence for use in the older age group [7-9,20,22]. In all five studies that included carotid imaging, older patients were significantly less likely to receive a scan than younger patients. One consequence of this finding is that older patients would not be consideration for endarterectomy, despite the evidence of significant benefit for older patients [30].

Stroke clinical guidelines generally provide recommendations for care to be applied to stroke patients of all ages [15]. However, unlike the recommendation for universal care within a stroke unit and carotid imaging, many other recommendations and process indicators are not supported by clear evidence for use in old age. As a consequence there is often uncertainty about best-practice interventions for older patients. Brain imaging is a useful example. In our review this was the most commonly reported process of care which was unsurprising given that the decision to deliver one of the few evidence based acute stroke therapies (thrombolytic therapy within 4.5 hours of stroke) relies on rapid imaging to exclude haemorrhagic stroke. In all studies that included brain imaging, older patients were less likely to receive the recommended care. This finding may reflect the fact that pivotal studies on the safe implementation of thrombolysis excluded patients older than 80 years, making clear recommendations about thrombolytic therapy for patients over 80 years difficult [5]. The lack of supporting evidence for this oldest group is also problematic for other process indicators in reviewed studies, such as antihypertensive therapy [31] and anticoagulation therapy [32].

The inconsistency of age-related findings in our review suggests that age-bias needs to be considered in terms of younger as well as older stroke sufferers. To illustrate, in one study inferior use of antihypertensive medications in younger patients compared to older patients was found [22]. Two studies also found that younger patients had inferior access to swallow screening compared to older patients [7,22], while a third study by McNaughton et al reported the opposite [19]. It should be noted that the McNaughton study used a small sample size and rated poorly on CASP quality scoring. More detailed, methodologically sound investigations are required to determine why age-related differences may occur, and why care decisions might differ between age groups and settings.

Several researchers nominated age discrimination as an important social determinant of health and proposed that systematic or covert age bias is largely to blame for inequitable care [7,33]. If this is so, there is unlikely to be a simple explanation in some aspects of stroke management. More research is needed which includes the oldest age groups and considers the complex factors which accompany ageing. It is also essential to improve our understanding of health discipline differences in clinical priorities and decision making, differences in hospital systems, staff knowledge of ageing, social determinants of care decisions and the impacts of broader cultural expectations. These complex influences remain poorly understood and inadequately investigated in stroke populations.

## Conclusions

Patients' age may be an important determinant of the stroke care they receive in acute settings. The literature demonstrated sub-optimal compliance with many evidence-based processes of care for older stroke patients compared to younger patients, including receiving CT head scans and carotid imaging. Conversely, younger patients appear to be disadvantaged in receiving antihypertensive medication. Data extracted from the included studies did not allow us to determine why these apparent inequities exist, or why care may differ between settings.

## Competing interests

The authors declare that they have no competing interests.

## Authors' contributions

JL conceived of the study, participated in study design, conducted the database search and systematic review, assessment of bias, analysis and synthesis of data, and drafted the manuscript. KGS supervised study design, participated in analysis of data, assisted in drafting and editing of the manuscript. KW participated in literature review, assessment of bias and editing the manuscript. JB participated in study design, drafting and editing of the manuscript. IE participated in study design, and editing of the manuscript. All authors read and approved the final manuscript.

## Declaration of sources of funding

The first author undertook this research as part of a PhD candidature which was supported by an Australian Postgraduate Award government scholarship. No other sources of funding were provided.

## Pre-publication history

The pre-publication history for this paper can be accessed here:

http://www.biomedcentral.com/1472-6963/11/161/prepub

## Supplementary Material

Additional file 1 **Search strategy MEDLINE (OVID)**. This is an example of the search strategy used in one database. Click here for file

Additional file 2 **Critical appraisal scores using the CASP Cohort Study appraisal tool**. Detailed scoring of the appraisal of bias is provided for each included study. Click here for file

Additional file 3 **All acute care process indicators examined by included studies**. A comprehensive list of all 56 process indicators is mapped against the literature in which they are reported. This included process indicators that are not evidence-based. Click here for file

Additional file 4 **Significant age-related differences in quality of acute stroke care for all process indicators**. A comprehensive list of all the statistically significant results for care adherence to all 56 process indicators. Results are mapped against the literature in which they are reported. Click here for file
