# Supplementary material for: Patients' age as a determinant of care received following acute stroke: A systematic review
Source: BMC Health Serv Res. 2011 Jul 6;11:161. doi: 10.1186/1472-6963-11-161 (PMC3150246; doi:10.1186/1472-6963-11-161)
Supplement: Additional file 1 — Search strategy MEDLINE (OVID). This is an example of the search strategy used in one database. [file 1472-6963-11-161-S1.DOC]

**Additional file 1 - Search strategy MEDLINE (OVID)**

1. Stroke/
2. “Quality of Health care”/
3. Total Quality Management/
4. Management Quality Circles/
5. Quality Control/
6. Quality Assurance, Health care/
7. Quality Indicators, Health care/
8. “Process Assessment (Health care)”/
9. "Outcome and Process Assessment (Health Care)"/
10. Quality Indicators, Health Care/
11. 2 or 3 or 4 or 5 or 6 or 7 or 8 or 9 or 10
12. 11 and 1
13. limit 12 to (english language and yr="1995 -Current")
