# Supplementary material for: Patients' age as a determinant of care received following acute stroke: A systematic review
Source: BMC Health Serv Res. 2011 Jul 6;11:161. doi: 10.1186/1472-6963-11-161 (PMC3150246; doi:10.1186/1472-6963-11-161)
Supplement: Additional file 2 — Critical appraisal scores using the CASP Cohort Study appraisal tool. Detailed scoring of the appraisal of bias is provided for each included study. [file 1472-6963-11-161-S2.DOC]

**Additional file 2 - Critical appraisal scores using the CASP Cohort Study appraisal tool**

| **CASP criteria** | Bhalla, 2004 (8) | Di Carlo, 1999 (9) | Fairhead, 2006 (20) | Heidrich, 2007 (21) | McKevitt, 2005 (11) | McNaughton, 2003 (19) | Palnum, 2008 (10) | Rudd, 2007 (7) | Saposnik, 2009 (22) |
| --- | --- | --- | --- | --- | --- | --- | --- | --- | --- |
| 1. Study addresses a clearly focussed issue | 1 | 1 | 1 | 1 | 1 | 1 | 1 | 1 | 1 |
| 1. Appropriate method used to answer the question | 1 | 1 | 1 | 1 | 1 | 0 | 1 | 1 | 1 |
| 1. Cohort recruitment was acceptable | 1 | 1 | 1 | 1 | 1 | 1 | 1 | 1 | 1 |
| 1. Exposure accurately measured to minimise bias | 1 | 1 | 1 | 1 | 1 | 1 | 1 | 1 | 1 |
| 1. Outcome accurately measured to minimise bias | 0 | 0 | 1 | 0 | 0 | 0 | 1 | 1 | 1 |
| 1. All important confounding factors identified and accounted for in the design &/or analysis | 0 | 0 | 0 | 1 | 0 | 0 | 0 | 0 | 1 |
| 1. Adequate follow-up of subjects | 1 | 1 | 1 | 0 | 1 | 1 | 1 | 0 | 0 |
| 1. Reporting of the results | 1 | 0 | 0 | 1 | 1 | 1 | 1 | 1 | 1 |
| 1. Precision of the results | 0 | 0 | 0 | 1 | 1 | 0 | 1 | 1 | 1 |
| 1. Believability of the results | 1 | 1 | 1 | 1 | 1 | 0 | 1 | 1 | 1 |
| 1. Transferability of the results | 0 | 0 | 0 | 0 | 0 | 0 | 0 | 0 | 0 |
| 1. Fit of results with other available evidence | 1 | 1 | 1 | 1 | 1 | 1 | 1 | 1 | 1 |
| **Total CASP score** (possible 12) | **8** | **7** | **8** | **9** | **9** | **6** | **10** | **9** | **10** |
