# Supplementary material for: Patients' age as a determinant of care received following acute stroke: A systematic review
Source: BMC Health Serv Res. 2011 Jul 6;11:161. doi: 10.1186/1472-6963-11-161 (PMC3150246; doi:10.1186/1472-6963-11-161)
Supplement: Additional file 3 — All acute care process indicators examined by included studies. A comprehensive list of all 56 process indicators is mapped against the literature in which they are reported. This included process indicators that are not evidence-based. [file 1472-6963-11-161-S3.DOC]

**Additional file 3 - All acute care process indicators examined by included studies**

| **Process of stroke care** | Palnum 2008 (10) | Rudd 2007 (7) | Bhalla 2004 (8) | Di Carlo 1999 (9) | Fairhead 2006 (20) | Heidrich 2007 (21) | McNaughton 2003 (19) | Saposnik 2009 (2) | McKevitt  2005 (11) |
| --- | --- | --- | --- | --- | --- | --- | --- | --- | --- |
| **Initial assessments and treatment** | | | | | | | | | |
| CT head scan performed | **Y** | **Y** | **Y** | **Y** |  | **Y** | **Y** |  | **Y** |
| MRI brain imaging performed | **Y** |  |  |  |  | **Y** |  |  | **Y** |
| Angiography performed |  |  | **Y** | **Y** | **Y** |  |  |  |  |
| Eye movements recorded |  | **Y** |  |  |  |  |  |  |  |
| Conscious level recorded |  | **Y** |  |  |  |  |  |  |  |
| Blood glucose level on arrival |  |  |  |  |  |  |  | **Y** |  |
| Thrombolysis therapy |  |  |  |  |  |  |  | **Y** |  |
| Asprin/antiplatelet therapy commenced early | **Y** | **Y** |  |  |  |  |  |  |  |
| Swallow screen /assessment |  | **Y** |  |  |  |  | **Y** | **Y** |  |
| Visual field assessment |  | **Y** |  |  |  |  |  |  |  |
| Sensory assessment |  | **Y** |  |  |  |  |  |  |  |
| Physiotherapy assessment/ physiotherapy | **Y** | **Y** |  |  |  |  |  | **Y** |  |
| Speech pathology communication assessment |  | **Y** |  |  |  |  |  |  |  |
| Occupational therapy assessment | **Y** | **Y** |  |  |  |  |  |  |  |
| Nutritional risk assessment | **Y** | **Y** |  |  |  |  |  |  |  |
| **Management and rehabilitation** | | | | | | | | | |
| Treatment in a stroke unit | **Y** | **Y** | **Y** |  |  |  |  | **Y** |  |
| Organised stroke care provided |  |  | **Y** |  |  |  |  | **Y** | **Y** |
| Multidisciplinary team meeting |  |  |  |  |  |  | **Y** |  |  |
| Pre-stroke function recorded |  | **Y** |  |  |  |  |  |  |  |
| Social work assessment |  | **Y** |  |  |  |  |  |  |  |
| Mood assessment |  | **Y** |  |  |  |  |  |  |  |
| Cognitive assessment |  | **Y** |  |  |  |  |  |  |  |
| Weight recorded |  | **Y** |  |  |  |  |  |  |  |
| Echocardiography performed |  |  | **Y** | **Y** |  | **Y** |  |  |  |
| Carotid imaging |  | **Y** | **Y** | **Y** | **Y** |  |  | **Y** |  |
| Surgical interventions (carotid & neurosurgery) |  |  |  | **Y** |  |  |  |  |  |
| Adequate clinical formulation |  | **Y** |  |  |  |  | **Y** |  |  |
| Doctor’s time per patient |  |  | **Y** |  |  |  |  |  |  |
| Nursing time per patient |  |  | **Y** |  |  |  |  |  |  |
| Rehabilitation therapy time per patient |  |  | **Y** | **Y** |  |  |  |  |  |
| Rehabilitation goals agreed by team |  | **Y** |  |  |  |  |  |  |  |
| Rehabilitation by physiotherapy/occupational therapy |  |  |  |  |  |  |  |  | **Y** |
| Rehabilitation by speech therapy |  |  |  |  |  |  |  |  | **Y** |
| Patient’s goals set regarding higher level functioning |  | **Y** |  |  |  |  |  |  |  |
| Urinary continence plan |  | **Y** |  |  |  |  |  |  |  |
| Complication prevention plan (positioning, handling) |  | **Y** |  |  |  |  |  |  |  |
| Complication prevention plan (DVT) |  | **Y** |  |  |  |  |  |  |  |
| Discussion about diagnosis, prognosis with patient |  | **Y** |  |  |  |  |  |  |  |
| Discussion about therapy goals with patient |  | **Y** |  |  |  |  |  |  |  |
| Discussion about diagnosis, prognosis with carer |  | **Y** |  |  |  |  |  |  |  |
| Discussion about therapy goals with carer |  | **Y** |  |  |  |  |  |  |  |
| **Secondary prevention** | | | | | | | | | |
| Blood cholesterol documented |  | **Y** |  |  |  |  |  |  |  |
| Dietary advice to reduce fat intake |  | **Y** |  |  |  |  |  |  |  |
| Other risk factors discussed with patient/carer |  | **Y** |  |  |  |  |  |  |  |
| Antithrombotic therapy initiated for ischemic stroke | **Y** | **Y** |  |  |  |  |  | **Y** |  |
| Anticoagulant therapy initiated for atrial fibrillation | **Y** |  |  |  |  |  |  | **Y** |  |
| Antihypertensive therapy at discharge |  |  |  |  |  |  |  | **Y** |  |
| Lipid regulating agent initiated |  | **Y** |  |  |  |  |  | **Y** |  |
| **Discharge planning** | | | | | | | | | |
| Transfer to a rehabilitation hospital |  |  | **Y** |  |  |  |  |  |  |
| Function at discharge recorded |  | **Y** |  |  |  |  |  |  |  |
| Patient/carer know discharge & follow-up plans |  | **Y** |  |  |  |  |  |  |  |
| Carer’s needs for support assessed separately |  | **Y** |  |  |  |  |  |  |  |
| Carer skills taught |  | **Y** |  |  |  |  |  |  |  |
| Home visit performed |  | **Y** |  |  |  |  |  |  |  |
| GP provided with timely information of discharge/death |  | **Y** |  |  |  |  |  |  |  |
| GP summary includes level of function at discharge |  | **Y** |  |  |  |  |  |  |  |

**Legend: Y** = process compliance examined by authors **Empty cell** = process compliance not examined
