# Supplementary material for: Patients' age as a determinant of care received following acute stroke: A systematic review
Source: BMC Health Serv Res. 2011 Jul 6;11:161. doi: 10.1186/1472-6963-11-161 (PMC3150246; doi:10.1186/1472-6963-11-161)
Supplement: Additional file 4 — Significant age-related differences in quality of acute stroke care for all process indicators. A comprehensive list of all the statistically significant results for care adherence to all 56 process indicators. Results are mapped against the literature in which they are reported. [file 1472-6963-11-161-S4.DOC]

**Additional file 4 - Significant age-related differences in quality of acute stroke care for all process indicators**

| **First author, year** | **Results** |
| --- | --- |
| Bhalla, 2004  (8) | Brain CT scans used more in patients aged <75 years (87%) than those >75years (79%) (*p<0.001).*  Angiography used more in patients aged <75years (11%) than those >75 years (1%) (*p<0.001).*  Echocardiography used more in patients aged <75years (34%) than those >75years (17%) (*p<0.001).*  Doppler used more in patients aged <75yrs (41%) than those >75yrs (22%) (*p<0.001).*  Access to organised stroke care higher for patients aged > 75 years (58%) than younger ones (51%) *(p=0.002).*  Nursing time higher for patients aged > 75years (3.7 mean hours/day, SD 1.2) than those < 75 years (3.6 mean hours/day, SD 1.3) (*(p=0.01).*  Rehabilitation therapy time less for patients aged > 75years (1.7 mean hours/day, SD 1.5) than those < 75 years (1.9 mean hours/day, SD 1.5) (*(p=0.03).*  Transfer to a rehabilitation hospital occurred less for patients aged > 75years (13%) than those < 75 years (17%) *(p=0.006).* |
| Di Carlo, 1999  (9) | Brain imaging used more in patients aged <80 years (87.7%) than those >80 years (66.9%) (*p<0.001).*  Carotid doppler imaging used more in patients aged <80 years (45.9%) than those >80 years (21%) (*p<0.001).*  Echocardiogram used more in patients aged <80 years (32.5%) than those >80 years (13.1%) (*p<0.001).*  Angiography used more in patients aged <80 years (10.3%) than those >80 years (0.9%) (*p<0.001).*  Neurosurgery performed more in patients aged <80 years (4.2%) than those >80 years (0.4%) (*p<0.001).*  Carotid surgery performed more in patients aged <80 years (1.2%) than those >80 years (0.2%) (*p=0.001).*  Speech therapy sessions were less for patients aged > 80 years (8.3 mean sessions +9.8) than those < 80 years (9.9 mean sessions +10) *(p=0.045).*  Occupational therapy sessions were less for patients aged > 80 years (9.1 mean sessions +10.5) than those < 80 years (11.8 mean sessions +11.5) *(p=0.004).* |
| Fairhead, 2006  (20) | Investigation of carotid stenosis lower for patients > 80 years than those <80 years (RR 0.36; 95% CI 0.28 – 0.46, *p< 0.0001)* |
| Heidrich, 2007  (21) | Brain CT scans used more in patients aged <74 years than those aged 75- 84 years (OR 0.5; 95% CI 0.3-0.8) or patients aged > 84 years (OR 0.3; CI 0.2-0.4). |
| McKevitt, 2005 (11) | Compared to patients aged <65 years, CT/MRI brain scan less likely for patients aged 75 – 84 years (OR0.36; 95% CI 0.20-0.67) and even less likely for patients older than 84 years (OR 0.15; 95% CI 0.08-0.30).  Compared to patients aged <65 years, care in a stroke unit more likely for patients aged 65 – 74 years (OR1.52; 95% CI 1.07-2.17); however no significant difference found for patients older than 74 years.  Patients younger than 65 years were less likely to get physiotherapy/occupational therapy rehabilitation than patients 65-74 years (OR 3.78; 95% CI 1.76-8.12), or patients 75-84 years (OR 2.15; 95% CI 0.98-4.69) or those older than 84 years (OR 4.24; 95% CI 1.22-14.73). |
| McNaughton, 2003 (19) | Swallow assessment was occurred more for patients age <75 years (30.2%) than those >75 (16.8%) *(p =0.03).*  CT brain scan occurred more for patients age <75 years (95.3%) than those >75 (72.6%) *(p <0.001).*  Adequate clinical formulation occurred more for patients aged <75 years (41.9%) than those >75 (27.4%) *(p =0.004).* |
| Palnum, 2008  (10) | Brain imaging with CT scan or MRI occurred more for patients < 65 years (83.5%) than those 65 – 80 years (79.4%; RR 0.95; 95% CI 0.93 – 0.96) or those >80 years (78.1%; RR 0.93; 95% CI 0.92- 0.95).  Treatment /rehabilitation in a stroke unit occurred more for patients < 65 years (77.1%) than those 65 – 80 years (76.5%; RR 0.99; 95% CI 0.98 -1.01) or those >80 years (74.9%; RR 0.97; 95% CI 0.95-0.99).  Oral anticoagulant therapy occurred more for patients< 65 years (66.1%) than those 65-80 years (60%; RR 0.91; 95% CI 0.82 - 0.99) or those >80 yrs (43.8%; RR 0.66; 95% CI 0.60 – 0.73).  Antiplatelet therapy occurred more for patients< 65 years (76.8%) than those 65-80 years (73.7%; RR 0.96; 95% CI 0.94 - 0.98) or those >80 yrs (70.2%; RR 0.91; 95% CI 0.89 – 0.94).  Assessment by a physiotherapist occurred more for patients < 65 years (50.2%) than those >80 yrs (47%; RR 0.94; 95% CI 0.90 – 0.97).  Assessment by an occupational therapist occurred more for patients < 65 years (44.8%) than those >80 yrs (42.3%; RR 0.95; 95% CI 0.91 – 0.98).  Nutritional risk evaluation occurred more for patients < 65 years (54.3%) than those 65- 80 years (49.4%; RR 0.91; 95% CI 0.88 - 0.94) or those >80 yrs (42.7%; RR 0.78; 95% CI 0.76 – 0.82). |
| Rudd 2007 (7) | This study provided risk adjusted predictions for care compliance with specified processes of care.  Older people less likely to be treated in a stroke unit than younger patients (risk ratio comparing 85+ years with those <65 years. RR 0.82 (95% CI 0.75 -0.90).  Brain scan <24 hours of stroke more likely for stoke unit patients <65 years (73%) than those 65-74 years (63%), 75-84 years (58%) or those aged > 85 years (59%). (Risk difference *P<0.001*)  Brain scan <24 hours of stroke more likely for non-stoke unit patients <65 years (73%) than those 65-74 years (63%), 75-84 years (59%) or those aged > 85 years (50%).(Risk difference *P<0.001*)  Eye movements recorded <24 hours more likely for stroke unit patients <65 years (82%) than those 75-84 years (78%) or those aged > 85 years (77%). (Risk difference *P<0.001*)  Eye movements recorded <24 hours more likely for non-stroke unit patients <65 years (71%) than those 75-84 years (64%) or those aged > 85 years (57%). (Risk difference *P<0.001*)  Swallow screen recorded <24 hours less likely for non-stroke unit patients <65 years (50%) than those 75-84 years (55%). (Risk difference *P<0.001*)  Formal assessment of visual fields <24 hours more likely for stroke unit patients <65 years (73%) than those aged > 85 years (65%). (Risk difference *P<0.001*)  Formal assessment of visual fields <24 hours more likely for non-stroke unit patients <65 years (63%) than those 65-74 years (59%), 75-84 years (54%) or those aged > 85 years (48%). (Risk difference *P<0.001*)  Formal assessment of sensory testing <24 hours more likely for stroke unit patients <65 years (83%) than those aged > 85 years (74%). (Risk difference *P<0.001*)  Formal assessment of sensory testing <24 hours more likely for non-stroke unit patients <65 years (72%) than those aged 75-84 years (64%) or those > 85 years (57%). (Risk difference *P<0.001*)  Clear diagnostic description made of cerebral lesion <24hours more likely for stroke unit patients <65 years (72%) than those aged > 85 years (66%). (Risk difference *P<0.001*)  Clear diagnostic description made of cerebral lesion <24hours more likely for non-stroke unit patients <65 years (62%) than those 65-74 years (55%), 75-84 years (52%) or those aged > 85 years (45%). (Risk difference *P<0.001*)  Commenced aspirin < 24hours more likely for stroke unit patients <65 years (75%) than those aged > 85 years (69%). (Risk difference *P<0.001*)  Commenced aspirin < 24hours more likely for non-stroke unit patients <65 years (68%) than those 65-74 years (64%), 75-84 years (60%) or those aged > 85 years (56%). (Risk difference *P<0.001*)  Speech & language therapist swallow assessment < 72 hours less likely for stroke unit patients <65 years (70%) than those 65-74 years (77%). (Risk difference *P<0.001*)  Speech & language therapist swallow assessment< 72 hours less likely for non-stroke unit patients <65 years (51%) than those 75-84 years (60%). (Risk difference *P<0.001*)  Speech & language therapist communication assessment < 7 days less likely for stroke unit patients <65 years (77%) than those 65-74 years (84%). (Risk difference *P<0.001*)  Physiotherapist assessment <72 hours of admission less likely for non-stroke unit patients <65 years (46%) than those 75-84 years (53%). (Risk difference *P<0.001*)  Occupational therapist assessment <7 days of admission more likely for stroke unit patients <65 years (68%) than those aged > 85 years (60%). (Risk difference *P<0.001*)  Nutritional needs assessment less likely for non-stroke unit patients <65 years (66%) than those 75-84 years (77%) or those aged > 85 years (77%). (Risk difference *P<0.001*)  Patients weight recorded less likely for non-stroke unit patients <65 years (37%) than those 75-84 years (42%). (Risk difference *P<0.001*)  Social work assessment <7 days of referral less likely for non-stroke unit patients <65 years (41%) than those 75-84 years (50%) or those aged > 85 years (51%). (Risk difference *P<0.001*)  Cognitive status assessment less likely for stroke unit patients <65 years (73%) than those 65-74 years (79%). (Risk difference *P<0.001*)  Cognitive status assessment less likely for non-stroke unit patients <65 years (48%) than those 65-74 years (53%), 75-84 years (57%) or those aged > 85 years (61%). (Risk difference *P<0.001*)  Individualised goals reference higher level functioning, more likely for stroke unit patients <65 years (52%) than those 65-74 years (42%), 75-84 years (35%) or those aged > 85 years (29%). (Risk difference *P<0.001*)  Individualised goals reference higher level functioning, more likely for non-stroke unit patients <65 years (25%) than those 75-84 years (16%) or those aged > 85 years (13%). (Risk difference *P<0.001*)  Plan to promote urinary continence more likely for stroke unit patients <65 years (75%) than those aged > 85 years (64%). (Risk difference *P<0.001*)  Plan to prevent post stroke complication (positioning & handling) more likely for stroke unit patients <65 years (93%) than those 75-84 years (89%) or those aged > 85 years (88%). (Risk difference *P<0.001*)  Plan to prevent post stroke complication (DVT) more likely for stroke unit patients <65 years (80%) than those 75-84 years (75%) or those aged > 85 years (74%). (Risk difference *P<0.001*)  Documented measure of blood cholesterol more likely for stroke unit patients <65 years (86%) than those aged > 85 years (80%). (Risk difference *P<0.001*)  Documented measure of blood cholesterol more likely for non-stroke unit patients <65 years (72%) than those 75-84 years (67%) or those aged > 85 years (65%). (Risk difference *P<0.001*)  Received dietary advice to reduce fat intake more likely for stroke unit patients <65 years (50%) than those aged > 85 years (37%). (Risk difference *P<0.001*)  Received dietary advice to reduce fat intake more likely for non-stroke unit patients <65 years (46%) than those 65-74 years (36%), 75-84 years (35%) aged > 85 years (29%). (Risk difference *P<0.001*)  On lipid regulating agent more likely for stroke unit patients <65 years (95%) than those aged > 85 years (83%). (Risk difference *P<0.001*)  On lipid regulating agent more likely for non-stroke unit patients <65 years (92%) than those 75-84 years (86%) or those aged > 85 years (77%). (Risk difference *P<0.001*)  Discussion of other risk factors with patient/carer more likely for stroke unit patients <65 years (69%) than those 65-74 years (58%), 75-84 years (46%) or those aged > 85 years (38%). (Risk difference *P<0.001*)  Discussion of other risk factors with patient/carer more likely for non-stroke unit patients <65 years (52%) than those 65-74 years (38%), 75-84 years (32%) or those aged > 85 years (23%). (Risk difference *P<0.001*)  Discussion about diagnosis with patient more likely for stroke unit patients <65 years (81%) than those 75-84 years (75%) or those aged > 85 years (71%). (Risk difference *P<0.001*)  Discussion about diagnosis with patient more likely for non-stroke unit patients <65 years (69%) than those 65-74 years (64%), 75-84 years (57%) or those aged > 85 years (53%). (Risk difference *P<0.001*)  Discussion about prognosis with patient more likely for stroke unit patients <65 years (78%) than those 75-84 years (70%) or those aged > 85 years (69%). (Risk difference *P<0.001*)  Discussion about prognosis with patient more likely for non-stroke unit patients <65 years (57%) than those 75-84 years (47%) or those aged > 85 years (45%). (Risk difference *P<0.001*)  Discussion about diagnosis with carer less likely for stroke unit patients <65 years (70%) than those 65-74 years (75%) 75-84 years (80%) or those aged > 85 years (84%). (Risk difference *P<0.001*)  Discussion about diagnosis with carer less likely for non-stroke unit patients <65 years (61%) than those 65-74 years (68%) 75-84 years (72%) or those aged > 85 years (78%). (Risk difference *P<0.001*)  Discussion about prognosis with carer less likely for stroke unit patients <65 years (69%) than those 65-74 years (76%) 75-84 years (80%) or those aged > 85 years (85%). (Risk difference *P<0.001*)  Discussion about prognosis with carer less likely for non-stroke unit patients <65 years (57%) than those 65-74 years (64%) 75-84 years (68%) or those aged > 85 years (75%). (Risk difference *P<0.001*)  Discussion about therapy goals with carer less likely for stroke unit patients <65 years (65%) than those 65-74 years (71%) 75-84 years (75%) or those aged > 85 years (76%). (Risk difference *P<0.001*)  Discussion about therapy goals with carer less likely for non-stroke unit patients <65 years (42%) than those 65-74 years (52%) 75-84 years (53%) or those aged > 85 years (60%). (Risk difference *P<0.001*)  Carer’s need for support assessed separately less likely for non-stroke unit patients <65 years (31%) than those aged 65-74 years (38%). (Risk difference *P<0.001*)  Home visit less likely for non-stroke unit patients <65 years (47%) than those 75-84 years (58%) or those aged > 85 years (62%). (Risk difference *P<0.001*)  GP informed of discharge (same day) or death (next day) more likely for stroke unit patients <65 years (70%) than those aged > 85 years (64%). (Risk difference *P<0.001*)  Discharge letter to GP includes level of function at discharge is less likely for non-stroke unit patients <65 years (42%) than those aged 75-84 years (50%). (Risk difference *P<0.001*)  Carotid imaging to check for stenosis more likely for stroke unit patients <65 years (71%) than those 65-74 years (63%), 75-84 years (51%) or those aged > 85 years (33%). (Risk difference *P<0.001*)  Carotid imaging to check for stenosis more likely for non-stroke unit patients <65 years (55%) than those 65-74 years (46%), 75-84 years (34%) or those aged > 85 years (16%). (Risk difference *P<0.001*) |
| Saposnik 2009  (22) | Older patients were more likely to have a swallow assessment than younger ones (45.7% of those < 59 years; 52.9% of 60-69 years; 59.2% of 70-79 years; 60.3% of 80+ years; *P<0.0001)*  Older patients were more likely to be discharged on antihypertensive drugs than younger ones (55.6% of those <59 years; 80.6% of 60-69 years; 80.5% of 70+ years; *P<0.001)*  Older patients less likely to have carotid imaging before discharge than younger ones (85.3% of those <59 years; 83.1% of 60-69 years; 81.8% of 70-79 years; 68.7% of 80+ years*; P<0.0001)* |
